# BIS targeting induces cellular senescence through the regulation of 14-3-3 zeta/STAT3/SKP2/p27 in glioblastoma cells

**DOI:** 10.1038/cddis.2014.501

**Published:** 2014-11-20

**Authors:** J-J Lee, J-S Lee, M N Cui, H H Yun, H Y Kim, S H Lee, J-H Lee

**Affiliations:** 1 Department of Biochemistry, College of Medicine, The Catholic University of Korea, Seoul, Korea; 2 Catholic Cancer Research Institute, College of Medicine, The Catholic University of Korea, Seoul, Korea; 3 Department of Biomedical Sciences, College of Medicine, Inha University, Incheon, Korea; 4 Cancer Evolution Center, College of Medicine, The Catholic University of Korea, Seoul, Korea

## Abstract

Cellular senescence is an important mechanism for preventing tumor progression. The elevated expression of Bcl-2-interacting cell death suppressor (BIS), an anti-apoptotic and anti-stress protein, often correlates with poor prognosis in several cancers including glioblastoma; however, the role of BIS in the regulation of senescence has not been well defined. Here, we describe for the first time that the depletion of BIS induces G1 arrest and cellular senescence through the accumulation of p27 that is independent of p53, p21 or p16. The increase in p27 expression in BIS-depleted cells was attributable to an impairment of the ubiquitin-mediated degradation of p27, which was caused by a decrease in S-phase kinase-associated protein 2 (SKP2) at the transcriptional level. As an underlying molecular mechanism, we demonstrate that the loss of activity of signal transducer and activator of transcription 3 (STAT3) was specifically linked to the suppression of SKP2 expression. Despite a reduction in phospho-STAT3 levels, total STAT3 levels were unexpectedly increased by BIS depletion, specifically in the insoluble fraction. Our results show that 14-3-3*ζ* expression is decreased by BIS knockdown and that 14-3-3*ζ* depletion *per se* significantly induced senescence phenotypes. In addition, the ectopic expression of 14-3-3*ζ* blocked senescence caused by BIS depletion, which was paralleled with a decrease in insoluble STAT3 in A172 glioblastoma cells. These findings indicate that the impairment of the protein quality control conferred by BIS and/or 14-3-3*ζ* is critical for BIS depletion-induced senescence. Moreover, BIS knockdown also induced senescence along with an accumulation of total STAT3 and p27 in several different cell types as well as embryonic fibroblasts derived from *Bis*-knock out mice with/without variations in 14-3-3*ζ* levels. Therefore, our findings suggest that a downregulation of BIS expression could serve as a potential strategy for restricting tumor progression via an induction of senescence through the regulation of STAT3/SKP2/p27 pathway.

Emerging evidence has shown that the induction of senescence, an irreversible cell growth arrest, could function as a tumor-suppressive mechanism to restrict tumor expansion.^1, 2^ However, frequent mutations in and subsequent functional inactivations of key regulators of cell cycle progression, such as p53, p21 or p16, confer tumor cells with the ability to bypass senescence, leading to oncogenic transformation.^3, 4^ Thus, the activation of senescence program that is not dependent upon the classical senescence pathway, involving p53–p21 or pRB–p16 signaling, could contribute to an increase in the therapeutic efficacy of chemotherapy or radiotherapy.^5, 6^

S-phase kinase-associated protein 2 (SKP2) is an F-box protein that functions as a substrate recognition unit of the Skp1-Clu1-F-box ubiquitin ligase complex.^7, 8^ Although SKP2 targets numerous cell cycle regulators for ubiquitination and degradation, the oncogenic potential of SKP2 is mainly linked to p27 degradation, as evidenced by low levels of p27 in aggressive tumors in which SKP2 expression is high.^9, 10, 11, 12, 13^ Furthermore, the inactivation of SKP2 through the regulation of abundance or activity has been shown to restrict tumorigenicity concomitantly with p27 accumulation.^5, 14, 15, 16^ In addition, the downregulation or loss of SKP2 is specifically associated with several senescence responses, most of which are p53 and p16 independent.^17, 18, 19, 20^ Given the inverse relationship between SKP2 and p27 levels, the regulation of SKP2–p27 axis warrants investigation as a critical determinant for cellular fate, especially in regard to restoring the senescence program in tumor cells in which p53 and/or p16 are defective. Recently, several studies have provided clues that link signal transducer and activator of transcription 3 (STAT3) signaling with SKP2–p27 axis. In colorectal cancer cells, the downregulation of STAT3 increases p27 expression.^21^ Subsequently, it has been reported that IL-6 or JAK2-mediated cell proliferation or invasion is due to an induction of the *SKP2* gene through STAT3 binding to the *SKP2* promoter.^22, 23, 24^ In addition, the anticancer effects of salinomycin in ovarian cancer cells were shown to be linked to the inhibition of STAT3 activity, which subsequently decreased SKP2 and increased p27 levels.^25^ Although these previous results indicate that *SKP2* is a direct target of STAT3, the regulatory function of STAT3 in SKP2/p27-induced senescence has not been previously clarified.

Accumulating evidence has shown that Bcl-2-interacting cell death suppressor (BIS) is an important molecule that sustains oncogenic characteristics of tumor cells. This is primarily based on its prominent pro-survival activity against various stresses *in vitro* and the observed overexpression of BIS in various types of human cancers including thyroid, prostate, pancreatic cancers and glioma.^26, 27, 28^ The mechanisms by which BIS regulate apoptotic process appear to be primarily based on its interaction with other apoptosis-regulating proteins such as BCL-2, IKK-*γ*, BAX or MCL-2, in cooperation with HSP70, thereby facilitating or inhibiting their degradation process or affecting their translocation within cellular compartments. ^26, 28, 29, 30, 31, 32^ Moreover, BIS, also known as BAG3, has been implicated in the invasive and metastatic phenotypes of cancers, suggesting a possible role as a prognostic marker.^33, 34, 35, 36^ Although the anti-apoptotic function of BIS appears to largely contribute to tumor expansion and resistance to chemotherapy, the impact of BIS on the inhibition of senescence and subsequent tumor progression has not been elucidated.

In this study, we demonstrate for the first time that BIS depletion results in a significant induction of premature senescence in several types of cells via a p27-dependent mechanism. We also identify the upstream signals involving 14-3-3*ζ*/STAT3/SKP2 pathways. Our findings have crucial implications for the targeting of BIS in therapeutic strategies to restrict the proliferation of cancer cells, especially for tumor cells that harbor intact p27 but defective of p53–p21 or p16 pathways.

## Results

### Induction of cell growth arrest and senescence by BIS depletion in A172 glioblastoma cells through a p27-dependent pathway

**Figure 1:**
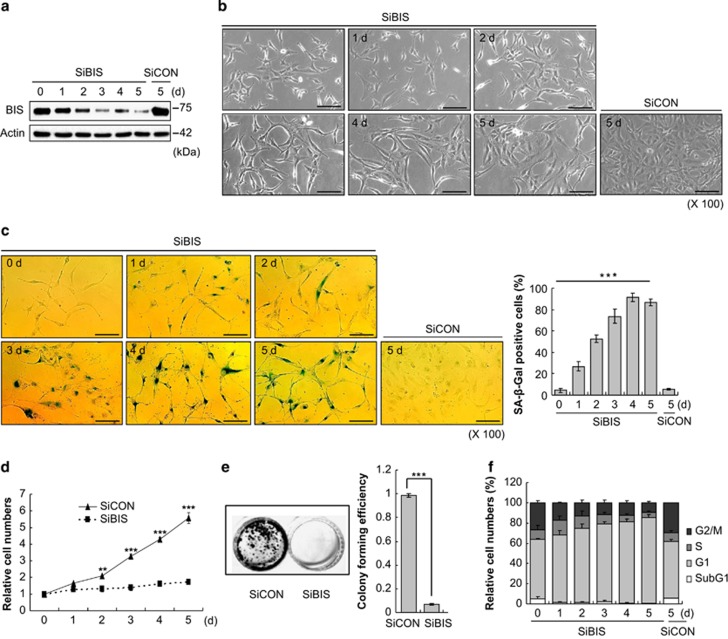
The depletion of BIS induces cell growth arrest and senescence in A172 glioblastoma cells. A172 cells were transfected with 100 nM of SiCON or SiBIS and incubated for the indicated times. d, day. (**a**) Western blot analysis for BIS expression. Actin levels are shown as a loading control. (**b**) Morphological changes and (**c**) SA-*β*-Gal staining and percentage (%) of SA-*β*-Gal-positive cells indicates that BIS depletion significantly induced senescence in A172 cells. ***P<0.001 *versus* day 0. Scale bars, 50 *μ*m. (**d**) Cell viability was determined by Trypan blue staining. The cell number at day 0 was designated as 1.0. **P<0.01, ***P<0.001 *versus* control cells. (**e**) Colony-forming ability was compared between SiBIS- and SiCON-treated cells at 14 days following transfection. The relative efficiency was presented in right column. ***P<0.001 *versus* SiCON-treated cells. (**f**) Cell cycle distribution was measured by flow cytometry at 5 days following transfection. Values are mean±S.E. of triplicate experiments

Using small interfering RNA (siRNA) strategy, BIS expression was efficiently suppressed in A172 glioblastoma cells; this suppression was sustained for up to 5 days following transfection, to 11.3% of control cells (Figure 1a). We then examined the influence of BIS depletion on cellular morphology, cell growth and apoptosis. Of note, cells treated with BIS-specific siRNA (SiBIS) showed typical senescence-related phenotypic changes in a time-dependent manner: large, flattened morphology and gradually increased senescence-associated *β*-galactosidase (SA-*β*-Gal) staining: 86.8% of cells were positive for SA-*β*-Gal staining at 5 days after transfection (Figures 1b and c). The proliferation rate was considerably slower in BIS knockdown cells compared in control cells as determined by relative increase in the cell numbers, 1.7-fold and 5.6-fold at day 5, respectively (Figure 1d). The colony-forming ability was also prominently suppressed in SiBIS-treated cells, by 92% compared with control siRNA (SiCON)-treated cells (Figure 1e). In addition, cell cycle profile demonstrated that the significant accumulation of cells in the G1 phase of the cell cycle accompanied by decrease in the cell populations in S or G2/M phase in SiBIS-treated cells, showing that the proportion of G1 phase was 84.7% and 59.9% in SiBIS- and SiCON-treated cells, respectively, at day 5 (*P*<0.05, Figure 1f). No obvious apoptosis resulted from BIS silencing as assessed by subG1 proportion, PARP cleavage and Annexin V staining, which was distinguished from the typical features of apoptosis induced by doxorubicin (Figure 1f and Supplementary Figures S1a–c). Collectively, BIS depletion leads to premature cellular senescence via G1 cell cycle arrest in A172 glioblastoma cells.

**Figure 2:**
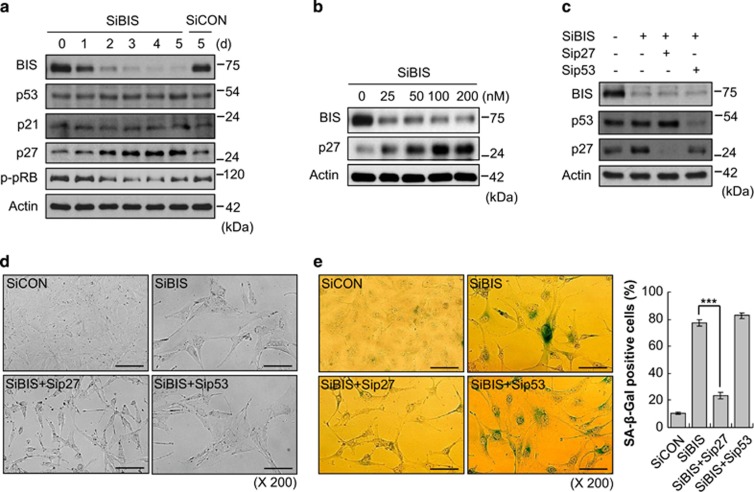
p27 is essential for BIS knockdown-induced senescence independent of the p53 and p21 pathway. (**a**) Following SiBIS (100 nM) treatment for the indicated times, the expression levels of BIS, p53, p21, p27 and p-pRB were examined by western blotting. The expression levels of each protein from SiCON-treated cells were provided for comparison. (**b**) p27 protein expression accumulated with increasing doses of SiBIS, as determined at 5 days following transfection. The effect of BIS depletion on p27 expression and the induction of senescence was restored by the co-transfection of siRNA for p27 (Sip27, 100 nM) but not by siRNA for p53 (Sip53, 100 nM) as determined by western blotting (**c**), an observation for morphological changes (**d**) and SA-*β*-Gal staining and percentage (%) of SA-*β*-Gal-positive cells (**e**). Bars represent mean±S.E. of triplicate experiments. ***P<0.001. Scale bars, 50 *μ*m

To further define the senescence process triggered by BIS depletion, we investigated activation status of several hallmarks of senescence following BIS knockdown, including p53, p21, p27 and pRB. Although p53 and p21 expression levels were not notably altered by BIS silencing, p27 protein levels were gradually increased and p-pRB levels were decreased in a time-dependent manner: 3.1-fold increase and 0.5-fold decrease, respectively, compared with those expression levels in the cells at day 0 (Figure 2a). The p27 levels were also progressively accumulated as increasing concentration of SiBIS (Figure 2b). As p16 has been known to be deficient in A172 cell lines,^37^ the decrease of p-pRB is likely due to the inactivation of CDK, which is affected by p27 but not by p16. To verify the potential involvement of p27 in BIS depletion-induced senescence, we examined the outcome of p27 or p53 siRNA transfection concomitantly with SiBIS. We found that the senescence-like morphologies and SA-*β*-Gal activities induced by SiBIS were reversed by p27 knockdown, from 77.9 to 23.5% as determined by SA-*β*-Gal-positive cells, but not by p53 knockdown (Figures 2c–e). These results indicate that p27 is essential for the induction of senescence by BIS silencing, which is independent of the p53–p21 axis.

### BIS modulates p27 protein stability *via* STAT3 and SKP2

**Figure 3:**
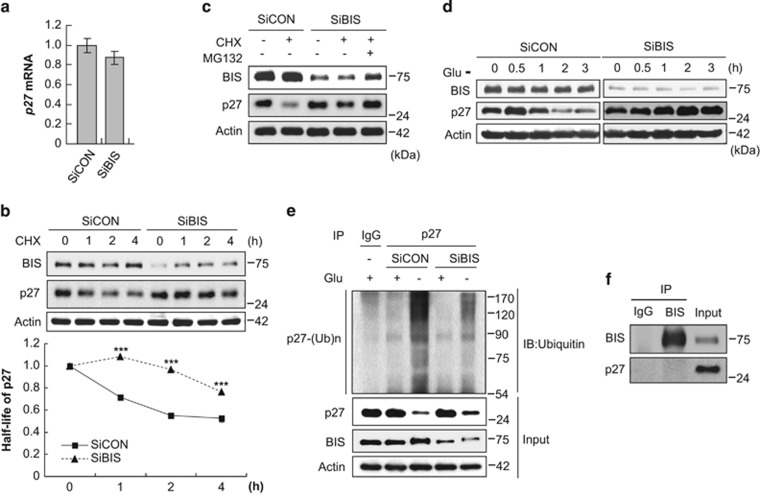
BIS modulates p27 levels via the regulation of 27 protein stability. (**a**) Relative mRNA levels for p27 were evaluated by qRT-PCR following SiCON or SiBIS (100 nM) treatment as described in the Materials and methods section. (**b**) p27 protein stability was examined by treatment of 40 *μ*g/ml CHX for the indicated times at 24 h post-transfection of SiCON or SiBIS. p27 protein levels from each time point were normalized to *β*-actin protein levels with Image J software and are presented as the mean value±S.E. from three independent experiments (lower column). ***P<0.001 *versus* control cells. h, hour. (**c**) Treatment of MG132 (10 *μ*M) for 1 h before CHX treatment for 2 h increased the stability of p27 in SiBIS-treated A172 cells. (**d**) The degradation of p27 caused by glucose (Glu)-free conditions was significantly prevented by BIS depletion. (**e**) The ubiquitination of p27 was decreased by BIS depletion. Two days following SiBIS transfection, cells were incubated in the presence or absence of glucose, and cell lysates were immunoprecipitated using p27 antibody or IgG, followed by immunoblotting with an ubiquitin (Ub) antibody. Total protein lysates were also subjected to western blot analyses with the indicated antibodies. (**f**) The interaction of endogenous BIS with p27 was investigated by immunoprecipitation of total cell lysates with BIS antibody and immunoblotted with p27 antibody

p27 expression is post-translationally regulated by proteasome-dependent degradation.^38^ In accordance with this, our results indicate that p27 mRNA levels are not significantly affected by BIS silencing as analyzed by quantitative real-time PCR (Figure 3a). Cycloheximide (CHX) chase experiments revealed that the degradation of p27 protein was notably delayed by BIS silencing compared with control cells (Figure 3b). p27 protein levels from CHX-treated cells were further accumulated about to two fold following pretreatment with MG132, a proteasome inhibitor, in BIS silencing cells (lanes 4 and 5 in Figure 3c), verifying that BIS depletion retarded the proteasome-dependent degradation of p27. Under glucose deprivation conditions in which p27 degradation was shown to be accelerated in a previous study,^39^ we confirmed that p27 protein is markedly stabilized by BIS depletion (Figure 3d). As shown in Figure 3e, glucose limitation significantly led to the poly-ubiquitination of p27 in control cells, which was suppressed by BIS depletion, showing an inverse relationship with cellular p27 levels. These data clearly indicate that BIS modulates p27 turnover through ubiquitin-mediated proteasomal degradation. To test whether interaction of BIS and p27 is involved in the degradation of p27, immunoprecipitation was performed, which demonstrated no direct interaction of BIS with p27 (Figure 3f).

**Figure 4:**
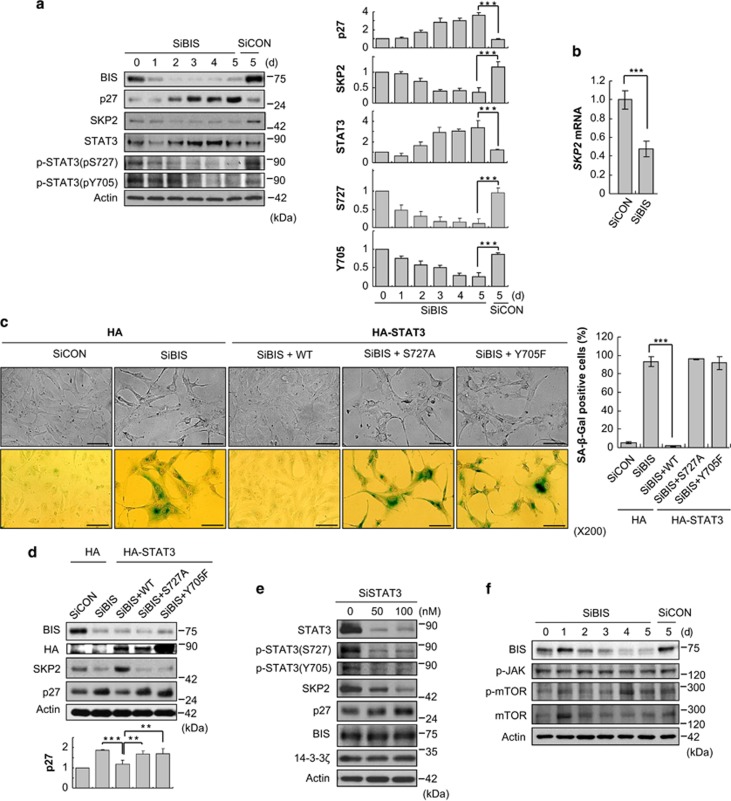
Inactivation of STAT3 is required for BIS knockdown-mediated senescence in A172 cells. (**a**) Immunoblotting for BIS, p27, SKP2, STAT3 and p-STAT3 (S727 and Y705) proteins in SiBIS (100 nM)-treated A172 cells. Densitometric analyses for each protein from three independent experiments were provided in lower column. ***P<0.001 *versus* the value from control cells. (**b**) Relative *SKP2* mRNA levels were evaluated by qRT-PCR. Bars represent mean±S.E. from three independent experiments. ***P<0.001. (**c**)The effects of STAT3 phosphorylation status on BIS silencing-mediated morphological changes and SA-*β*-Gal activities were assessed by the transfection of 1 *μ*g/ml of empty vector (HA), WT or STAT3 mutants (S727A and Y705F) concomitantly with SiCON or SiBIS (100 nM). Scale bars, 50 *μ*m. (**d**) Reversal of SKP2 and p27 levels after WT-STAT3 was provided by western blot assay. Densitometric analysis for p27 from three independent experiments was shown in lower column. **P<0.01 and ***P<0.001 between indicated groups. (**e**) The effects of STAT3 depletion on STAT3, p-STAT3 (S727 and Y705), SKP2, p27, BIS and 14-3-3*ζ* status were examined by western blotting. (**f**) No significant decreases in p-JAK, p-mTOR or mTOR expression levels were observed following after SiBIS transfection

As SKP2 is a major determinant of p27 levels,^7, 8, 10^ we examined the effects of BIS depletion on the expression of SKP2. Immunoblottig showed that SKP2 levels were prominently decreased as BIS decreased, to 42% of control cells at day 5, which was inversely correlated with p27 (Figure 4a). *SKP2* mRNA levels were also decreased to 45% of control cells at 3 days following SiBIS transfection (Figure 4b). This prompted us to investigate which transcription factor was responsible for the alteration of *SKP2* mRNA by BIS depletion. Recent studies have reported that STAT3 activation upregulates SKP2 expression, which leads to p27 degradation, in association with the survival and invasive ability of various cancer cells.^23, 24, 25^ Thus, we assessed whether the STAT3 signaling pathway was involved in the senescence phenotypes induced by BIS depletion. We found that the phosphorylation of STAT3, representing the activated form of STAT3 as a transcriptional regulator, was profoundly decreased by BIS depletion in a time-dependent manner as determined by immunoblotting using specific antibodies for phospho-STAT3 (p-STAT3) that target pS727 and pY705 (Figure 4a). To clarify whether the inactivation of STAT3 is critical for BIS-modulated senescence, dominant-negative STAT3 constructs and wild type-STAT3 (WT-STAT3) were introduced into A172 cells before BIS knockdown. The expression of WT-STAT3 almost completely prevented the senescence responses induced by BIS depletion, whereas the expression of two dominant-negative mutants, Y705F-STAT3 and S727A-STAT3, did not affect the senescence phenotype (Figure 4c). In agreement with the observed morphological changes, SKP2 expression was restored by WT-STAT3 in BIS-depleted cells, which led to a decrease in p27 protein levels; there was no effect from either inactive mutant (Figure 4d). Interestingly, however, the total STAT3 levels were not parallel with the levels of p-STAT3, rather significantly increased by BIS knockdown (Figure 4a). To address whether the accumulation of total STAT3 has an impact on senescence induction, we suppressed STAT3 expression using specific siRNA. As illustrated in Figure 4e, the depletion of STAT3 activation, accompanied with a decrease in active p-STAT3, both pY705 and pS727, downregulated SKP2 but increased p27 expression, comparable to what was observed following BIS depletion. These results indicate that decreased STAT3 activation is associated with the progression of senescence initiated by BIS knockdown.

### 14-3-3*ζ* is involved in the BIS-mediated regulation of the STAT3/SKP2/p27 pathway

The activation of STAT3 is primarily mediated by the phosphorylation of Y705, which is maximized or suppressed by phosphorylation at S727, resulting in the modulation of inflammation, survival, angiogenesis and motility.^40, 41^ Among the numerous kinases that mediate the phosphorylation of STAT3, JAK2 and mTOR were examined as the prototypical kinases for Y705 and S727, respectively.^41, 42, 43^ No detectable changes were observed in the phosphorylation status of JAK and mTOR following BIS silencing (Figure 4f). Thus, the decreased phosphorylation of STAT3 is not because of a disruption in upstream signals from JAK or mTOR.

**Figure 5:**
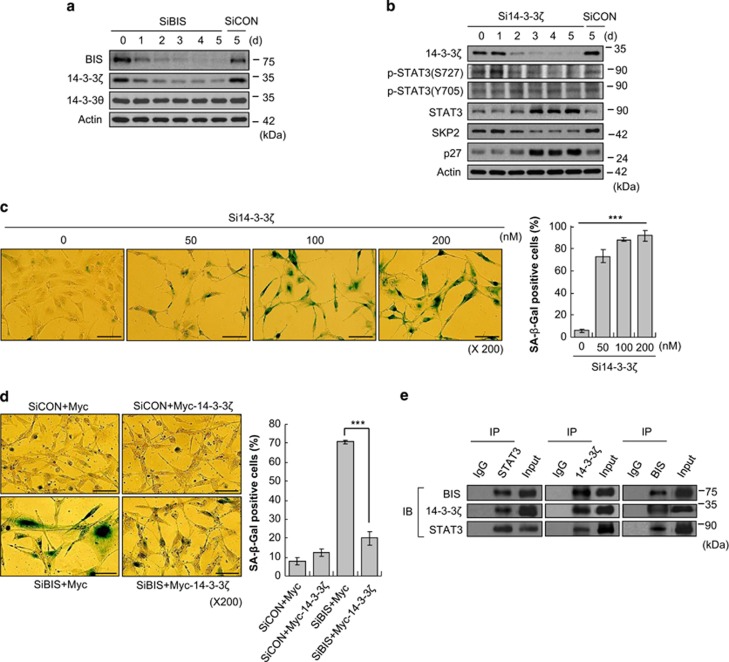
14-3-3*ζ* is involved in BIS depletion-induced senescence through the regulation of the STAT3 pathway. (**a**) Western blot assay for 14-3-3*ζ* and 14-3-3*θ* following SiBIS (100 nM) treatment. (**b**) The expression levels of 14-3-3*ζ*, p-STAT3 (S727 and Y705), STAT3, SKP2 and p27 were examined at the indicated times following 14-3-3*ζ* knockdown. (**c**) 14-3-3*ζ* depletion-induced senescence as determined by SA-*β*-Gal activities. ***P<0.001 *versus* control cells. (**d**) The overexpression of 14-3-3*ζ* prevented BIS depletion-induced senescence. Bars represent mean±S.E. from triplicate experiments.The percentage of SA-*β*-Gal-positive cells is shown in the right column. ***P<0.001 *versus* SiBIS-only treated cells. Scale bars, 50 *μ*m. (**e**) Immunoprecipitation and immunoblotting was performed with the indicated antibodies following the transfection of STAT3, 14-3-3*ζ* or BIS-expressing plasmid for 2 days

Recently, sequential systemic proteomic analyses demonstrated that BIS and STAT3 were enrolled in the lists of 14-3-3*ζ* interactome and, reversely, 14-3-3*ζ* was recognized as a BIS-binding protein in the BIS interactome.^44, 45^ The importance of 14-3-3*ζ* and BIS was described in promoting aggresome formation.^46^ Moreover, 14-3-3*ζ* was reported to interact with STAT3 in multiple myeloma cells.^47^ Based on these previous findings, we tested the possibility that 14-3-3*ζ* is involved in the coupling of BIS and STAT3 with respect to the regulation of senescence. Figure 5a shows a gradual decrease in 14-3-3*ζ* expression following the decrease in BIS, whereas the expression of 14-3-3*θ*, another 14-3-3 protein isoform, was not altered. We next evaluated if 14-3-3*ζ* silencing *per se* could trigger senescence. Immunoblotting analysis indicated that 14-3-3*ζ*-depleted cells exhibited similar profiles to BIS-depleted cells with regards to the expression of p-STAT3 (S727), total STAT3, SKP2 and p27, thus acting as a substitute for BIS depletion (Figure 5b). p-STAT3 (S727) levels but not p-STAT3 (Y705) were abolished upon 14-3-3*ζ* knockdown, which was in keeping with the previous report showing that 14-3-3*ζ* protects the dephosphorylation of S727 sites of STAT3 from PPA2 in multiple myeloma cells.^47^ In addition, the marked increase in the SA-*β*-Gal-positive cells was observed in 14-3-3*ζ*-depleted cells (Figure 5c). The SA-*β*-Gal assays also revealed that ectopic expression of 14-3-3*ζ* almost completely prevented senescence initiated by BIS depletion (Figure 5d). Taken together, these findings indicate that 14-3-3*ζ* expression is modulated by BIS and that 14-3-3*ζ* protein functions as a potent regulator of cellular senescence as well. Subsequent investigation into the physical association between BIS, 14-3-3*ζ* and STAT3 using immunoprecipitation and subsequent immunoblotting assays indicate that these proteins constitute one complex (Figure 5e).

### BIS and 14-3-3*ζ* modulate the solubility of STAT3

**Figure 6:**
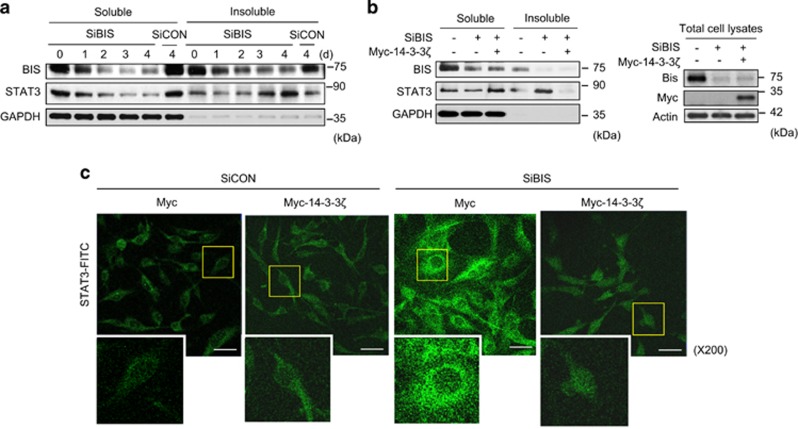
BIS downregulation increases aggregated STAT3, which is restored by 14-3-3*ζ*. (**a**) STAT3 protein solubility is decreased by BIS knockdown (SiBIS, 100 nM). Total cell lysates were separated into soluble and insoluble fractions as described in Materials and methods section and subjected to a western blot assay for STAT3. (**b**) The effects of 14-3-3*ζ* on the solubility of STAT3 protein levels were examined by transfection with Myc-14-3-3*ζ* (1 *μ*g/ml) with SiBIS and analyzed by western blot. The expression levels of BIS and 14-3-3*ζ* are verified in the right column. (**c**) Representative confocal immunofluorescence images analyzing endogenous STAT3 in A172 cells transfected with empty vector (Myc) or Myc-14-3-3*ζ* with SiCON or SiBIS. Endogenous STAT3 was detected using an anti-STAT3 antibody and FITC-conjugated anti-rabbit IgG. Lower panels represent higher magnifications of the selected area. Scale bars, 50 *μ*m

Both BIS and 14-3-3*ζ* proteins have been reported to share chaperon-like function that prevents protein aggregation and the ability that promotes the aggresomal targeting of unfolded proteins. ^46, 48, 49, 50, 51^ Thus, the increase of total STAT3 in both BIS-depleted cells (Figure 4a) and 14-3-3*ζ*-silenced cells (Figure 5b) raises the question of whether the loss of protein quality control (PQC) functions of BIS and 14-3-3*ζ* is involved in the accumulation of STAT3 aggregates, primarily nonfunctioning STAT3. We investigated the solubility of STAT3 by fractioning cell lysates into soluble and insoluble parts following BIS depletion. Notably, the total amount of STAT3 was reduced in soluble fraction, whereas it was increased in insoluble fraction after BIS silencing in a time-dependent manner; decrease to 0.19-fold in soluble fraction and increase to 2.36-fold in insoluble fraction at day 4 compared with those levels at day 0 (Figure 6a). To check whether 14-3-3*ζ* could prevent the accumulation of STAT3 in insoluble fraction as a result of BIS depletion, we performed fractionation experiments following Myc-14-3-3*ζ* overexpression before SiBIS treatment. Ectopic expression of 14-3-3*ζ* led to a substantial reversal of STAT3 solubility, shifting from insoluble to soluble fraction (Figure 6b). This reversal in STAT3 solubility was further illustrated by the observation of immunostaining using confocal analysis. A significant increase in the intensity and size of STAT3-positive dots, representing aggregated STAT3, in SiBIS-treated cells, was reduced by 14-3-3*ζ* overexpression accompanied by a reduction in cell size (Figure 6c). Altogether, these data strongly indicate that loss of the dis-aggregating function of BIS and/or 14-3-3*ζ* is responsible for STAT3 aggregation, which leads to the functional inactivation of STAT3.

### BIS is negative regulator of senescence in various types of cells

**Figure 7:**
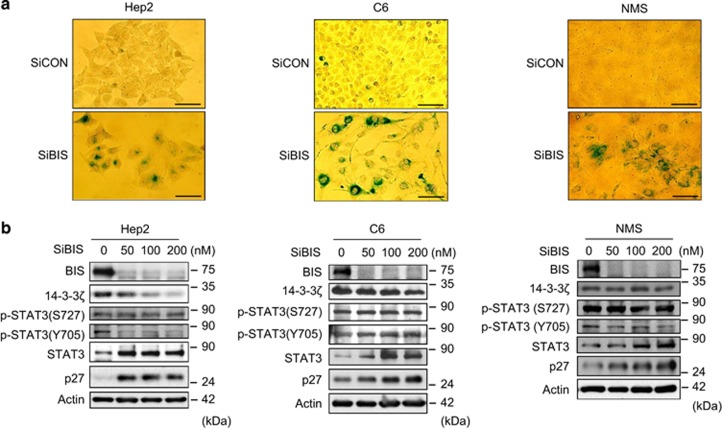
Loss of BIS induces cellular senescence in various types of cells including Hep2, C6 and NMS. (**a**) Representative images of SA-*β*-Gal staining in each of the cell lines examined at 4 days following SiBIS transfection (100 nM). Scale bars, 50 *μ*m. (**b**) Expression levels of BIS, 14-3-3*ζ*, p-STAT3 (S727 and Y705), STAT3, SKP2 and p27 were examined by immunoblotting from each of the cell lines

**Figure 8:**
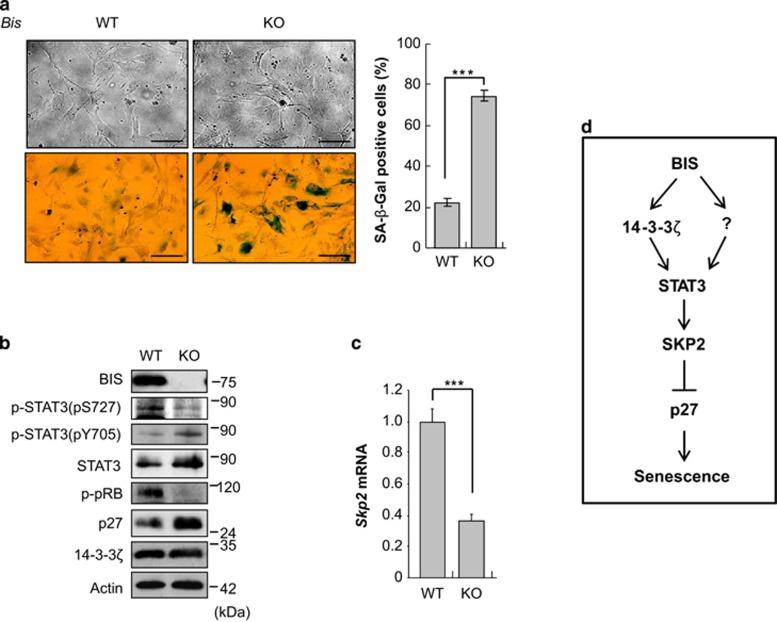
Serum limitation accelerates the induction of senescence in *Bis-*KO MEF cells. MEFs derived from *Bis-*WT or deficient mice were incubated with 10% FBS for 4 days. (**a**) Morphological changes and SA-*β*-Gal activities were evaluated. ***P<0.001. Scale bars, 50 *μ*m. (**b**) Expression levels of p-STAT3, STAT3, p-pRB, p27 and 14-3-3*ζ* were determined by immunoblotting. (**c**) *Skp2* transcript levels were determined by real-time PCR. ***P<0.001. (**d**) Proposed model summarizing how BIS depletion results in cellular senescence

We investigated the BIS depletion effect in other cell lines including C6 rat glioma cells, Hep2 human laryngeal cancer cells and NMS rat kidney cells. As with A172 cells, all of these cells showed senescence-like morphological changes and an increase in SA-*β*-Gal activity following BIS silencing (Figure 7a and Supplementary Figure S2a) and showed similar profiles of total STAT3 and p27 accumulation and decrease in *SKP2* mRNA (Figure 7b and Supplementary Figure S2b). However, 14-3-3*ζ* and p-STAT3 levels were not consistent across the cell types tested. We also examined the induction of senescence using mouse embryonic fibroblasts (MEFs) cells at passage 2 derived from WT and *Bis* gene-knock out (KO) mice; 22% and 74% cells were positive for SA-*β*-Gal staining, respectively, after incubation for 5 days in 10% serum (Figure 8a). The expression levels of p-STAT3 (S727), total STAT3 and p27 were increased and *Skp2* mRNA levels were decreased in *Bis*-KO MEF, whereas 14-3-3*ζ* and p-STAT3 (Y705) levels were not decreased (Figures 8b and c). Overall, these data suggest that BIS negatively regulates the induction of senescence in various types of cells, via STAT3/SKP2/p27 pathways, which involves 14-3-3*ζ* depending on the cellular context.

## Discussion

In this study, we demonstrate for the first time that BIS depletion results in the premature senescence of various types of cancer cells, including glioma cells, through p27-dependent and p53-independent pathway. The stabilization of p27 at the protein levels was attributed to SKP2 downregulation, which was driven by the loss-of-function of STAT3. We further demonstrate that the inactivation of STAT3 is linked to a decrease in its solubility, which was most likely regulated by PQC function of 14-3-3*ζ* through interactions with BIS. Based on our results, we propose a working hypothesis for BIS depletion-induced cellular senescence involving 14-3-3*ζ–*STAT3–SKP2–p27 signaling axis as summarized in Figure 8d. Our findings provide a therapeutic implication for BIS targeting-induced senescence in the repression in tumor growth in a variety of cancers in which BIS is highly expressed.

STAT3 is an important signaling node that is involved in multiple pathways including inflammation, differentiation, proliferation or angiogenesis through the activation of target genes following the translocation of p-STAT3. Although STAT3 activity has been shown to influence SKP2 expression and subsequently p27 levels in several cancer cells,^21, 22, 23, 24, 25^ it had remained whether STAT3-mediated SKP2/p27 regulation exhibited an important role in the direction of senescence program. In our study, the observed BIS depletion-mediated decrease in SKP2 was inversely correlated with p-STAT3 levels. Furthermore, the overexpression of WT-STAT3, but not non-phosphorylated STAT3, rescued glioblastoma cells from the induction of senescence combined with a restoration of SKP2 expression levels. This indicates functional inactivation of STAT3 is crucial in BIS deficiency-induced and SKP2/p27-mediated senescence. With regard to the senescence response, the role of STAT3 appears to be inconsistent and depends on the cellular context or condition. In normal fibroblasts and hepatic satellite cells, STAT3 activation is required for IL-6- or IL-22-induced senescence, through the expression of IGFBP5 or SOC3, respectively,^52, 53^ On the other hand, the inactivation of STAT3 was essential to induce cellular senescence program in breast, colon and lung cancer cells following DNA damage,^54, 55^ in accordance with our results. Thus, our findings demonstrating the critical involvement of STAT3 activity in SKP2/p27-mediated senescence may reinforce the significance of the crosstalk between STAT3 signaling and the SKP2/p27 pathway in tumor progression through the inhibition of senescence in addition to the promotion of proliferation or invasion.

Notably, we provide important evidence that maintenance of STAT3 solubility is critical for transcriptional activator activity of STAT3, in addition to the conventional upstream kinase activity. Cellular protein solubility or the maintenance of the proper protein folding structure in response to variety of cellular stresses is under the control of the PQC machinery. This system executes the re-solubilization or clearance of misfolded aggregating proteins and thereby prevents the accumulation of toxic protein aggregates.^56^ Our results show that total STAT3 levels were specifically increased in the insoluble fraction following BIS depletion in immunoblotting, supported by immunofluorescence observations. Thus, the increase in total STAT3 following BIS depletion represents a functionally inactive status and possibly aggregated forms of STAT3, leading to conformational changes that are unable to maintain a phosphorylated status at active sites. Our hypothesis is supported by previous studies using STAT3 inhibitor, demonstrating that a selective disruption of STAT3 dimerization resulted in its aggregation in perinuclear aggresomes, followed by a decrease in STAT3 phosphorylation and nuclear translocation.^57^ The regulatory function of BIS on STAT3 solubility is in line with previous findings indicating that BIS participates in PQC process to prevent the accumulation of misfolded proteins. BIS has been shown to enhance the degradation of proteins, which are prone to aggregation because of genetic mutation, such as *α*B-crystallin, superoxide dismutase and Huntingtin protein, by promoting the macroautophagic pathway or aggresomal targeting of substrates through interactions with HSP70, HSPB8, p62 or 14-3-3*γ*.^46, 58, 59, 60, 61^ The physiological significance for the role of BIS in protein homeostasis has been implicated *in vivo* by the upregulation of BIS–HspB8 complex in astrocytes of the human brain affected by protein aggregation diseases such as Huntington's diseases and spinocerebellar ataxia type 3.^62^ This indicates that the induction of BIS was driven by the necessity to facilitate the clearance of aggregated proteins within an aggregation-prone milieu. In our studies, BIS depletion itself could constitute an aggregation-prone environment for STAT3, resulting in cellular senescence. In other words, even without an aggregation-inducing stressor, the cellular abundance of BIS could affect the proper folding or solubility of specific proteins, of which deterioration changes in cell fate. Our results, therefore, strengthen the importance of the PQC, in which BIS and other key molecules function in concert, to protect cells from entering into senescence program as well as from neurodegenerative diseases.

In this study, we observed that BIS depletion was paralleled with a decrease in the level of 14-3-3*ζ*, which appeared to be an essential prerequisite for BIS depletion-mediated STAT3 aggregation and senescence in A172 cells. We also demonstrated that BIS, 14-3-3*ζ* and STAT3 proteins are present as a complex, consistent with previous proteomic analyses.^44^ Although it is not certain how BIS modulates 14-3-3*ζ* expression, it is possible that the interaction of BIS and 14-3-3*ζ* might confer stabilization of 14-3-3*ζ* protein, probably via protecting from proteasomal degradation, as previously suggested in the interaction with IKK*γ* or MCL-1.^30, 31^ Intriguingly, some of isoforms of 14-3-3, such as 14-3-3*γ* and 14-3-3*ζ*, share this anti-aggregating activity with BIS either through chaperone-like functions or through aggresome-promoting activities.^46, 48, 49, 50, 51^ Thus, the stable complex of BIS and 14-3-3*ζ* in turn might have a major role in the PQC for STAT3 in terms of preserving the native folding or solubility of STAT3. However, p-STAT3 (Y705) levels were not affected by 14-3-3*ζ* depletion, whereas BIS knockdown significantly decreased both p-STAT3, Y705 and S727, in A172 cells. Furthermore, the alterations of 14-3-3*ζ* levels or p-STAT3 levels were not consistently observed in NMS or MEF cells in which BIS depletion clearly induced senescence with an accumulation of total STAT3. Therefore, BIS might affect the proper folding or solubility of STAT3 through interaction with 14-3-3*ζ* or through another pathway unidentified. It is possible that BIS could directly interact with STAT3 for proper protein folding, as observed in the mutated in *α*B-crystallin.^61^ BIS was shown to interact only with dimers of 14-3-3*γ*, not with monomers.^46^ Therefore, it is also possible that, depending on the cellular context, different isoforms of the14-3-3 family might interact with BIS to maintain the solubility of specific target proteins. Alternatively, BIS depletion might affect the chaperone function of 14-3-3 through the disruption of the balance between monomeric and dimeric forms without altering 14-3-3 protein levels. The molecular mechanism, by which BIS and 14-3-3 proteins participate in PQC, either separately or in collaboration, warrants further extensive study.

In conclusion, we have shown that BIS depletion induces senescence through the regulation of STAT3/SKP2/p27 axis in various types of cells. Considering that little is known about the regulatory mechanism of p27-mediated senescence induction, our results describe a novel function of BIS, extending from anti-apoptotic or anti-stress roles to the negative regulator of p27-mediated senescence. Therefore, BIS targeting could be implicated as a therapeutic strategy for inducing senescence in the prevention of tumor progression, particularly those showing defect in p53 or p16 pathways.

## Materials and Methods

### Cell lines and reagents

A172 human glioblastoma cells, C6 rat glioma cells and Hep2 human laryngeal cancer cells were cultured in DMEM, and NMS rat kidney cells were cultured in RPMI supplemented with 10% fetal bovine serum (FBS, Thermo Fisher Scientific, Waltham, MA, USA) and 1% penicillin–streptomycin (Thermo Fisher Scientific). MEFs were prepared from *Bis*^+/+^ and *Bis*^−/−^ mouse embryos ^63^ and were grown in DMEM with 20% FBS before serum restriction. MG132 and CHX were purchased from Sigma-Aldrich (St. Louis, MO, USA). Doxorubicin was obtained from Calbiochem (San Diego, CA, USA). siRNA duplexes against BIS (5′-AAGGUUCAGACCAUCUUGGAA-3′), STAT3 (5′-CCAACGACCUGCAGCAAUA-3′), 14-3-3*ζ* (5′-CCAACGACCUGCAGCAAUA-3′), p53 (5′-CCACUUGAUGGAGAGUAUU-3′), and p27 (5′-AAGTACGAGTGGCAAGAGGTG-3′) and control duplex (5′-CCUACGCCACCAAUU UCGU-3′) were purchased from Bioneer Inc. (Daejeon, Korea). The 14-3-3*ζ* overexpression plasmid was cloned into the pCMV-Myc vector (Clonetech, Mountain View, CA, USA). The plasmids for WT and mutant forms of STAT3 (Y705F and S727A) in pCS2+ were obtained from Ira O Daar (National Cancer Institute, Frederick, MD, USA). The transfection of the indicated plasmids and siRNA were carried out using Fugene Extreme (Roche Diagnostics, Mannheim, Germany) and the Lipofectamine 2000 reagent (Invitrogen, Carlsbad, CA, USA), respectively.

### Cell morphology, SA-*β*-Gal staining and immunofluorescence

The morphological characteristics of cells were evaluated using an inverted phase contrast microscope (Olympus, Center Valley, PA, USA). For SA-*β*-Gal staining, we followed the protocol described in Dimri *et al.*^64^ For the immunofluorescence analyses, fixed cells were incubated with an anti-STAT3 monoclonal antibody, and immunoreactivity was visualized with fluorescein isothiocyanate (FITC)-conjugated anti-mouse IgG under a Zeiss LSM 700 laser fluorescence confocal microscope using Zen software (Carl Zeiss MicroImaging GHBH, Jena, Germany). Morphological examinations were performed at 4 days following each treatment, otherwise indicated.

### Colony formation analysis, cell cycle analysis and apoptotis assay

Colony formation ability was assessed by seeding A172 cells at a density of 500 cells per 60-mm dish and staining with crystal violet (Sigma-Aldrich) after 14 days. Colonies were counted using a colony counter (BioLogics, Manassas, VA, USA). Cell cycle distributions and apoptotic cells were evaluated by propidium iodide (50 *μ*g/ml) staining and a FITC-Annexin V apoptosis detection kit (BD Bioscience, San Jose, CA, USA), respectively. Data acquisition and analysis were performed in flow cytometer (FACSCalibur, BD Bioscience) using CellQuest Pro software (BD Bioscience).

### Western blot analysis

For western blot analysis, cells were lysed in RIPA buffer and centrifuged. Then, equal amounts of total protein were separated on a SDS-PAGE gel. After transferring the proteins to a nitrocellulose membrane, the membrane was blocked with 5% (w/v) nonfat dry milk in PBS overnight at 4 °C. After incubating with specific primary antibodies, the antibody–antigen complexes were visualized using horseradish peroxidase (HRP)-conjugated secondary antibodies and a standard chemiluminescence system (Thermo Fisher Scientific) according to the manufacturer's instructions. The soluble and insoluble fractions from total cell lysates were prepared as described in Yano M *et al.*^48^ p53 and p27 antibodies were purchased from Novocastra Inc. (Newcastle, UK) and BD Bioscience, respectively. Phospho-pRB, p-STAT3 (Y705 and S727), STAT3, SKP2, phospho-JAK, phospho-mTOR, mTOR and PARP antibodies were obtained from Cell Signaling Inc. (Danvers, MA, USA). Antibodies for p21, 14-3-3*ζ*, 14-3-3*θ*, GAPDH, *β*-actin, Myc, Ubiquitin, HRP-conjugated anti-rabbit, anti-mouse and FITC-conjugated anti-mouse were purchased from Santa Cruz Biotechnology (Santa Cruz, CA, USA). Quantification of the intensities of each band was carried out using Image J software, which is provided by the National Institute of Health (NIH, Bethesda, MD, USA). The relative densities for each protein were determined by normalizing with that of *β*-actin.

### Quantitative real-time PCR analysis

RNA was isolated with an RNA extraction kit AcuZol (Bioneer Inc.) and subjected to reverse transcription using M-MLV reverse transcriptase (ReverTra Ace qPCR RT Kit, Toyobo, Osaka, Japan) according to the manufacturer's protocol. Then, quantitative real-time PCR (qRT-PCR) was performed using SYBR premix Ex Taq (Takara Biotechnology, Shiga, Japan) with specific primers on Applied Biosystems 7300 PCR machine (Applied Biosystems, Carlsbad, CA, USA). The relative values for BIS, p27 and SKP2 mRNA were calculated after normalizing the Ct value to *β*-actin levels from the same sample using the ddCt method.

### Immunopreciptation

An equal amount of each protein lysate was incubated with the indicated antibodies, normal rabbit IgG or normal mouse IgG (Santa Cruz Biotechnology) for 4 h at 4 °C, followed by an incubation with 20 *μ*l of protein A magnetic beads (Millipore, Billerica, MA, USA) for 16 h at 4 °C. The immune complexes were analyzed by western blot analyses with the indicated antibodies. Protein lysates were also subjected to western blot analyses with the indicated antibodies.

### Statistics

Statistical values are expressed as the mean±S.E. Multiple comparisons between groups were assessed by one-way ANOVA with Bonferroni's correction (SPSS v. 11.5; IBM, Armonk, NY, USA). Probability values <0.05 were accepted as statistically significant.

## Abbreviations

BIS: Bcl-2-interacting cell death suppressor
SKP2: S-phase kinase-associated protein 2
STAT3: signal transducer and activator of transcription 3
SA-*β*-Gal: senescence-associated *β*-galactosidase
siRNA: small interfering RNA
WT: wild type
KO: knock out
PQC: protein quality control
