# Supplementary material for: BIS targeting induces cellular senescence through the regulation of 14-3-3 zeta/STAT3/SKP2/p27 in glioblastoma cells
Source: Cell Death Dis. 2014 Nov 20;5(11):e1537–. doi: 10.1038/cddis.2014.501 (PMC4260756; doi:10.1038/cddis.2014.501)
Supplement: Supplementary Figure S1 [file cddis2014501x1.ppt]

## Slide 1
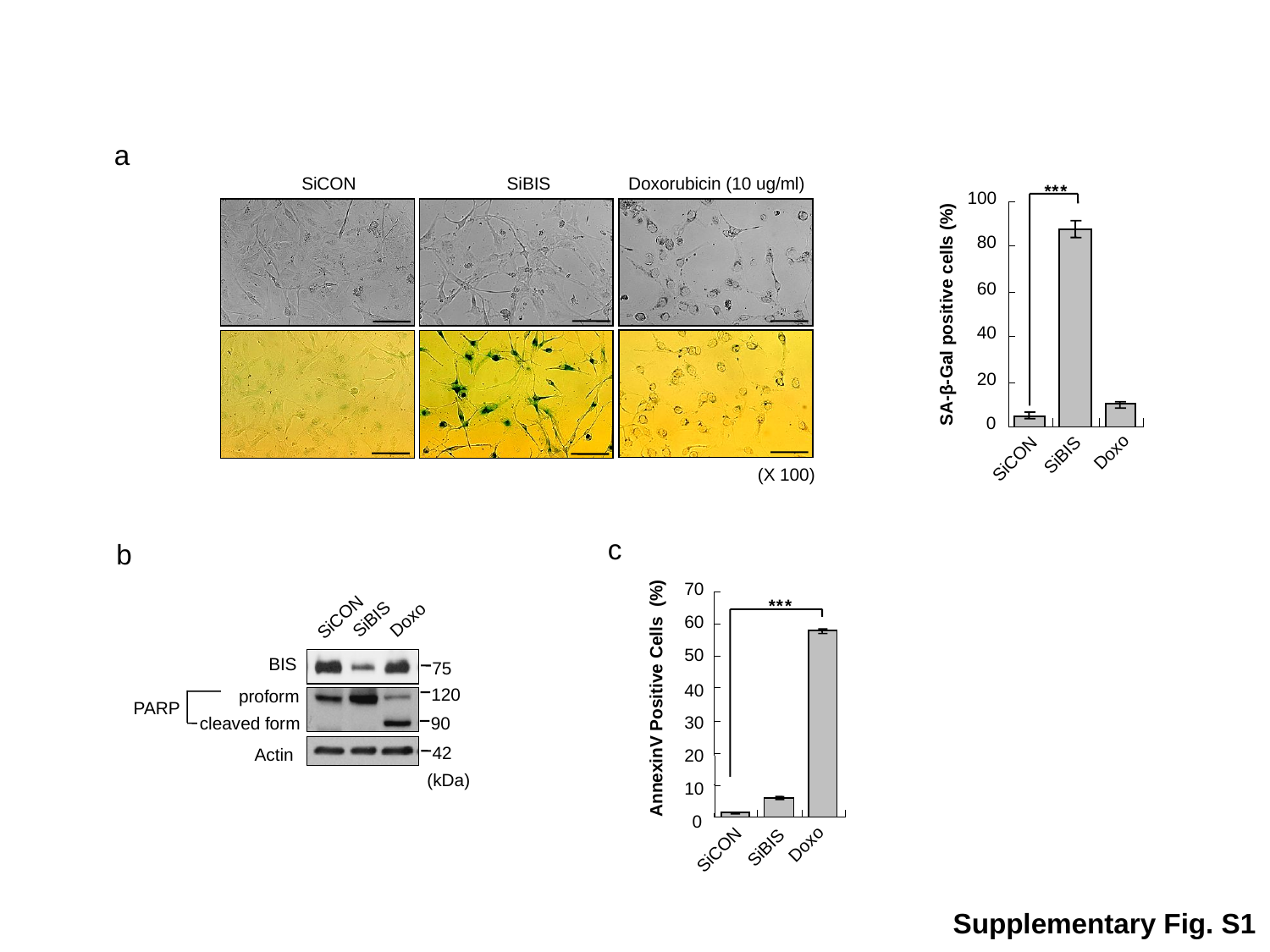

SA-β-Gal positive cells (%)
a
 SiCON SiBIS Doxorubicin (10 ug/ml)
(X 100)
*
*
*
100
80
60
40
20
0
Doxo
SiBIS
SiCON
AnnexinV Positive Cells (%)
c
SiBIS
Doxo
SiCON
BIS
75
120
proform
PARP
cleaved form
90
42
Actin
(kDa)
b
70
*
*
*
60
50
40
30
20
10
0
Doxo
SiBIS
SiCON
Supplementary Fig. S1
