# Supplementary material for: BIS targeting induces cellular senescence through the regulation of 14-3-3 zeta/STAT3/SKP2/p27 in glioblastoma cells
Source: Cell Death Dis. 2014 Nov 20;5(11):e1537–. doi: 10.1038/cddis.2014.501 (PMC4260756; doi:10.1038/cddis.2014.501)
Supplement: Supplementary Figure S2 [file cddis2014501x2.ppt]

## Slide 1
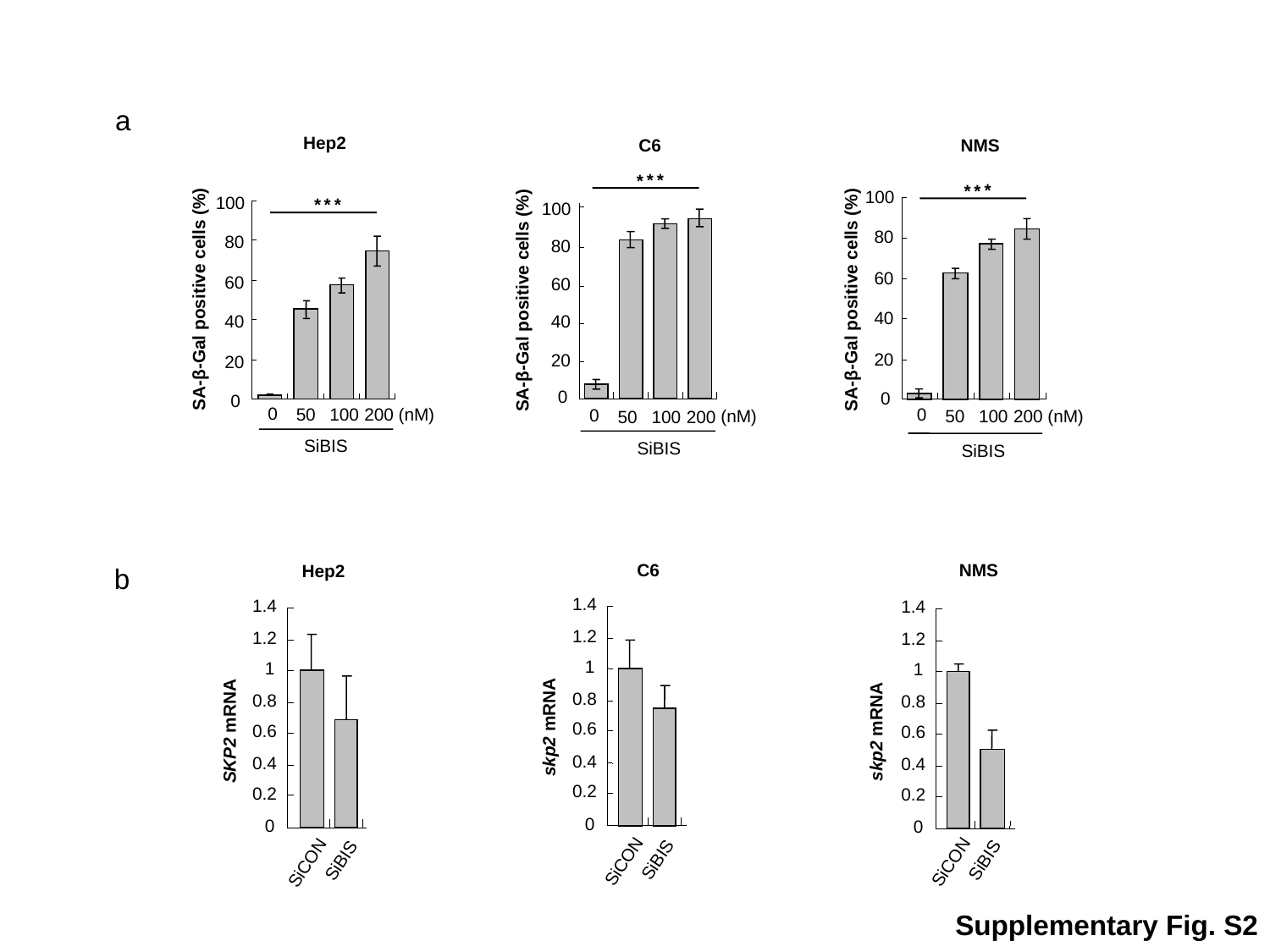

a
SA-β-Gal positive cells (%)
SA-β-Gal positive cells (%)
NMS
*
*
*
100
80
60
40
20
0
0
(nM)
50
100
200
SiBIS
SA-β-Gal positive cells (%)
C6
*
*
*
100
80
60
40
20
0
0
(nM)
50
100
200
SiBIS
Hep2
*
*
*
100
80
60
40
20
0
0
(nM)
50
100
200
SiBIS
skp2 mRNA
C6
1.4
1.2
1
0.8
0.6
0.4
0.2
0
SiBIS
SiCON
SKP2 mRNA
Hep2
1.4
1.2
1
0.8
0.6
0.4
0.2
0
SiBIS
SiCON
skp2 mRNA
NMS
1.4
1.2
1
0.8
0.6
0.4
0.2
0
SiBIS
SiCON
b
Supplementary Fig. S2
