# Supplementary material for: BIS targeting induces cellular senescence through the regulation of 14-3-3 zeta/STAT3/SKP2/p27 in glioblastoma cells
Source: Cell Death Dis. 2014 Nov 20;5(11):e1537–. doi: 10.1038/cddis.2014.501 (PMC4260756; doi:10.1038/cddis.2014.501)
Supplement: Supplementary Figure Legends [file cddis2014501x3.doc]

**Supplementary Figure S1** The effect of BIS SiRNA is distinguished from apoptosis. (a) The morphological changes and SA-β-Gal activities were examined at 4 days following 100 nM SiBIS transfection or at 2 days following10 μg/ml doxorubicin treatment. Cell death was examined by western blotting with cleaved PARP antibody (b) and Annexin-V assay (c). Bars represent mean ± SE from triplicate experiments. ***P<0.001. Scale bars, 50 μm

**Supplementary Figure S2** Loss of **Bis** increases SA-β-Gal activity and decreases *SKP2* mRNA levels in other cell lines. (a) SA-β-Gal activity was examined at 4 days in Hep2, C6 and NMS cells following transfection with different doses of SiBIS and the percentage of SA-β-Gal positive cells is presented as the mean ± SE from three independent experiments. ***P<0.001. (b) *SKP2* transcript levels from each of the cell lines were determined by qRT-PCR in SiCON or SiBIS-treated cells. The mean value from triplicate experiments was provided and the values from SiCON-treated cells were designated as 1.0

­­­­­­­­­­­­­­­­­­­­­­­­­­­
